# Ulcers of the fingers and dorsum of the left hand caused by venous hypertension after construction of a brachiocephalic arteriovenous fistula: case report

**DOI:** 10.1590/1677-5449.190008

**Published:** 2019-10-23

**Authors:** Aline Ioshie Akamine Asari, Daiane Cristina Ferreira Damasceno, Nathália Araújo de Almeida, Hícaro do Carmo Moreira, Ricardo André Viana Barros, Francisco Alberto Bezerra Ximenes

**Affiliations:** 1 Hospital de Base do Distrito Federal – HBDF, Unidade de Cirurgia Vascular, Brasília, DF, Brazil.

**Keywords:** arteriovenous fistula, renal dialysis, chronic renal failure, ulcer cutaneous

## Abstract

Venous ulcers caused by venous hypertension secondary to arteriovenous fistulae are rare. Their etiology can be confirmed by vascular Doppler ultrasonography, which can differentiate between stenosis of central vessels and hemodynamic overload caused by development of tributaries from the vein responsible for the arteriovenous fistula. We present a case caused by hemodynamic overload of a tributary, which diverted the primary flow from the fistula to the distal limb. We chose to ligate the fistula to treat the ulcers and create another arteriovenous fistula in the contralateral limb.

## INTRODUCTION

Venous hypertension of the hand caused by a hemodialysis arteriovenous fistula (AVF) in an upper limb is considered a rare complication. Case series in the literature report an incidence rate of 0.13 to 0.78%. Among these cases, occurrence of ulcers due to upper limb venous hypertension is rarer still, and Debus et al. found just 16 cases in the literature. Such ulcers can be caused by hemodialysis AVF, particularly if created by side-to-side anastomosis, or may be due to congenital arteriovenous malformation or traumatic vascular injury, which can be complicated by central vein stenosis or valve incompetence in the AVF drainage vein. Vascular echography is helpful for diagnosing etiology and to guide treatment, the objective of which is to reduce venous pressure, whether by construction of a bypass for venous drainage, complete reconstruction of the AVF, or ligature of the fistula.^1^^,^ 2

## CASE DESCRIPTION

**Figure 1:**
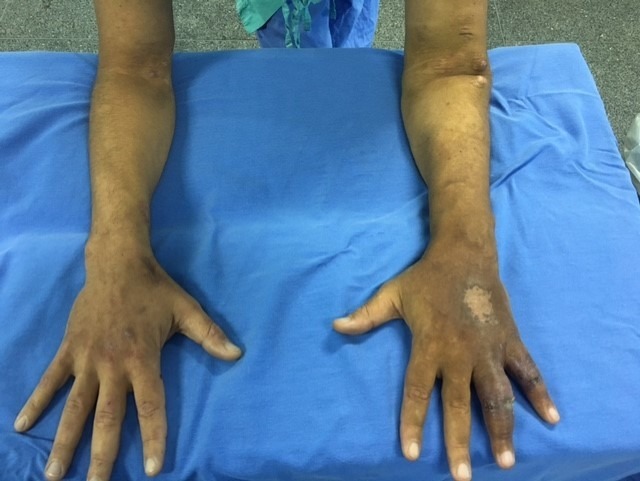
Upper limbs: edema and hyperpigmentation of dorsal surface of left hand.

**Figure 2:**
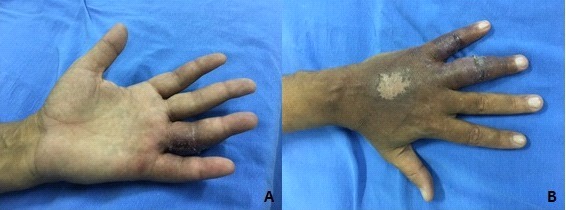
Left hand: (A) ulcer on the palmar surface of the fourth finger and (B) healed ulcer on the dorsal surface of the hand and the fourth and fifth fingers.

A 45-year-old male patient, formerly hypertensive, who had been involved in an automobile accident 17 years previously, was diagnosed with hemothorax and underwent thoracotomy and tracheostomy. He remained in the intensive care unit for 105 days, during which period he was given nephrotoxic antibiotics, and developed chronic renal failure. He had a brachiocephalic AVF in the left arm, which developed increased flow after 10 years, with ulcers on the fourth and fifth fingers and dorsum of the left hand (Figures 1 and 2), causing pain that limited manual activities.

**Figure 3:**
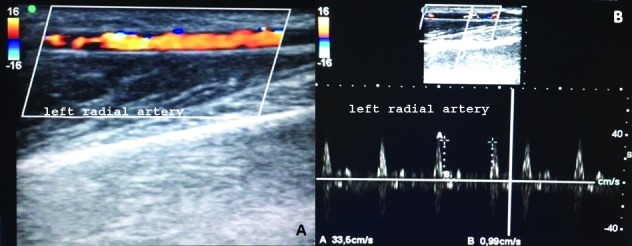
Left radial artery patent with triphasic flow. Peak systolic velocity of 33.3 cm/s.

Vascular Doppler ultrasonography of the AVF showed a patent anastomosis. Arterial flow was normal from the brachial artery to the radial and ulnar arteries (Figure 3) and flow direction, systolic and diastolic velocity, resistance indices and other echographic features were all incompatible with venous access steal syndrome.

**Figure 4:**
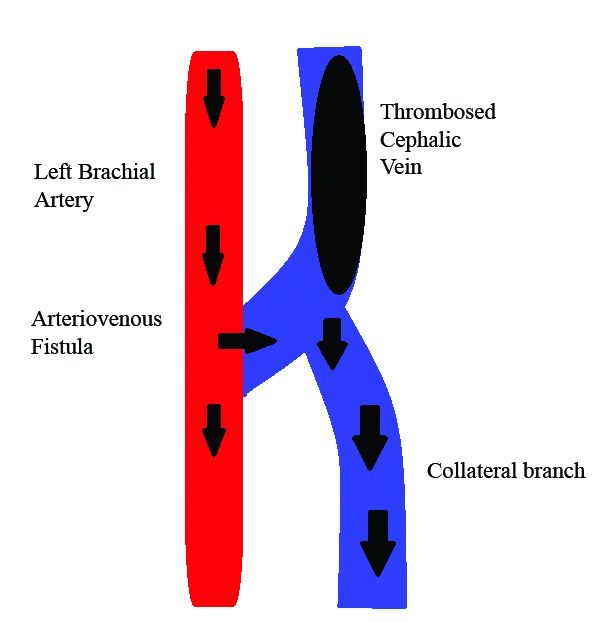
Diagram illustrating the left brachiocephalic arteriovenous fistula with cephalic vein thrombosis and flow diverted to the collateral branch.

**Figure 5:**
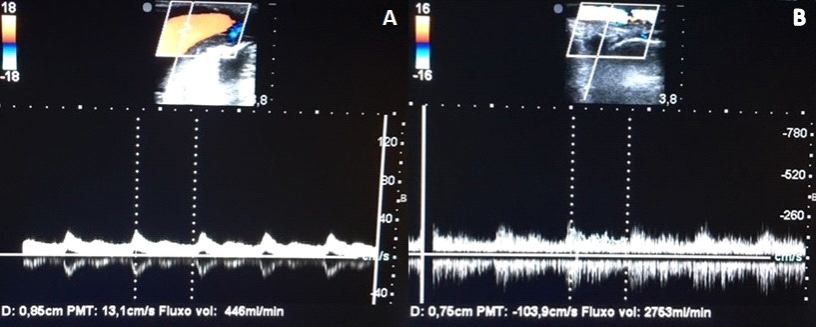
Flow volume through collateral branch: proximal (A) at 446 mL/min; distal and at the level of the wrist (B) at 2,763 mL/min.

The diagnosis made according to clinical signs and supplementary echographic details was therefore venous hypertension due to occlusion of the vein in the arm providing return (the cephalic) and overdevelopment of the descending collateral (Figure 4). The AVF flow diverted through the collateral branch was 446 mL/min, reaching 2,763 mL/min at the level of the wrist (Figure 5).

Having detected the complication in course, the patient underwent an examination with mapping to detect the best site to construct a new AVF, which was a superficialized right brachiobasilic fistula. The patient continued receiving dialysis via the AVF in the left upper limb until the new AVF had matured, and it was not necessary to insert a catheter.

**Figure 6:**
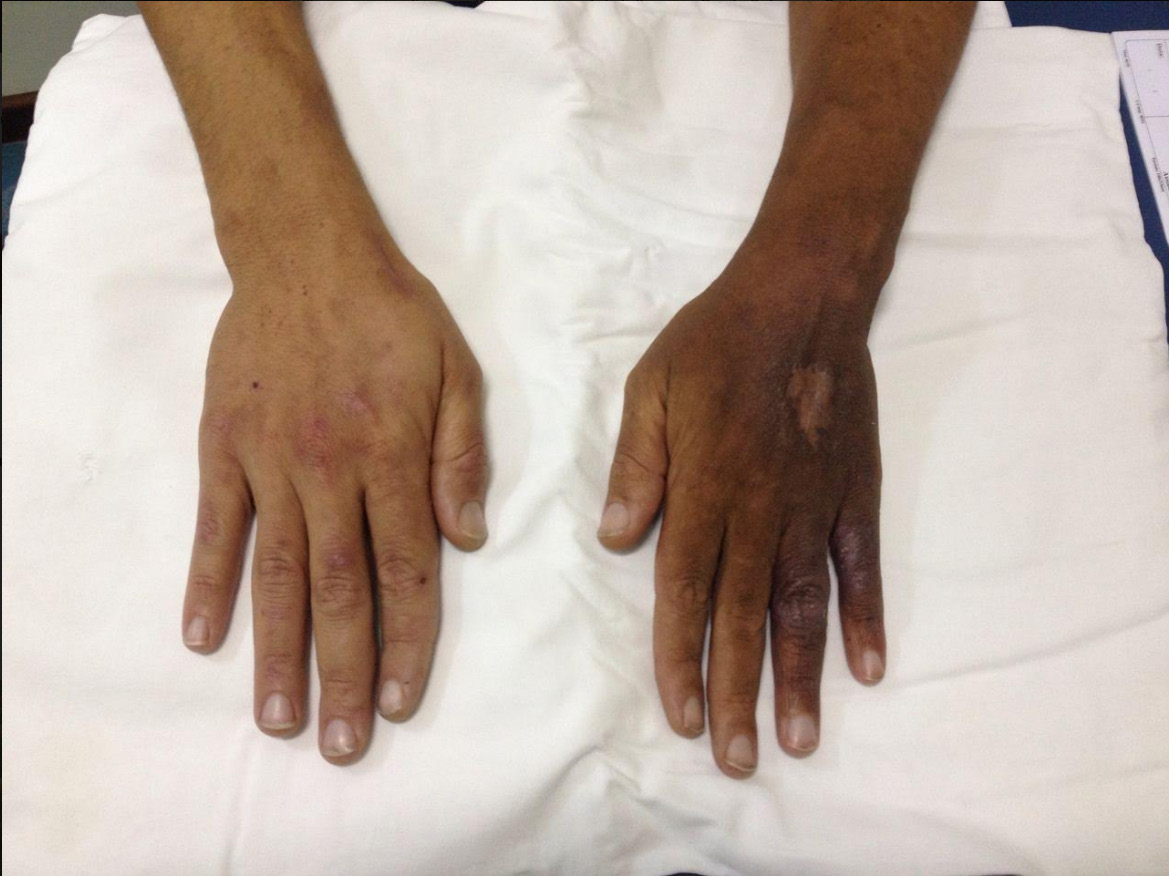
Appearance 2 weeks after ligature of the arteriovenous fistula.

After maturation of the AVF, the tributary in the left limb was ligated and sectioned at the most proximal point possible (in the elbow). Edema had improved and the finger ulcers started to heal within 1 week of the operation. The patient returned to the clinic in the second week, with the ulcers already healed. At follow-up, 2 months after discharge, function in the left hand had already been recovered (Figure 6). The right brachiobasilic fistula had good thrill and flow during hemodialysis, and only a darker coloration of the right limb remained.

## DISCUSSION

Venous hypertension is an important problem among hemodialysis patients, because it causes significant edema in the affected limb and compromises the AVF. It is one of the many possible long-term complications of creation of AVFs for hemodialysis. It develops for one of the following two reasons: as a consequence of stenosis of central vessels or because of hemodynamic overload caused by development of tributaries arising from the vein chosen for anastomosis with the artery.^3^

Similar to venous hypertension of the lower limbs, which can be sequelae of deep venous thrombosis or primary valve incompetence, it courses with symptoms that can sometimes cause limitations, such as edema and skin ulcerations, in addition to repulsion elicited by the esthetic appearance. In extreme cases, ulcerations can also be subject to secondary contamination, exacerbating the damage. When the upper limbs are involved, there can be limitations to daily manual activities and even implications for use of the ipsilateral vascular access.

The symptomology can be confused with vascular access steal syndrome, but vascular echography can be used for differentiation and to acquire details to plan the strategy for surgical repair.^3^ As described here, distal arterial supply to the affected limb was normal and, on echography, it was observed that the patient did not have perforating veins to the deep vein system arising from the principal descending tributary, which could amplify the signs of venous hypertension.

A study conducted by Nascimento and Riella investigating complications related to fistulae found that the most prevalent was thrombosis (80% of cases), which occurs because of stenosis, frequently of the venous anastomosis, due to intimal hyperplasia.^4^^,^ 5 In the case presented here, after occlusion of the cephalic vein in the arm, flow developed in a collateral, flowing from the elbow in the direction of the hand, and became the only puncture route and was useful for a long time. However, routine use of this collateral as puncture site led to development of venous hypertension with symptoms limiting the patient’s activities. This case illustrates the controversy about when and for how long collaterals should be used for hemodialysis access, since the smaller caliber makes complications, which involve the distal tissues, more pervasive.

In 1986, Currier et al.^6^ proposed a standard nomenclature for procedures related to AVFs and developed a classification of the degree of severity of venous hypertension, as follows: 0, zero severity; 1, mild, with minimal symptoms, discoloration and discrete edema of the extremity; 2, moderate, characterized by intermittent discomfort and severe edema, with intervention usually needed; and 3, severe, characterized by persistent discomfort with hyperpigmentation of the skin, persistent and severe edema and venous ulcers, with intervention mandatory.

The patient in this case was at grade 3 severity, with an unquestionable need for intervention. A new AVF was created, followed by ligature of the previous AVF. However, this sequence may not be feasible when there are infections of the ulcers caused by venous hypertension, and can even cause hematogenic infection via any catheters that may be needed. Bacteremia is a common problem among patients on hemodialysis, as much as 26 times more frequent than in the general population, and surgical exposure during infections is a dangerous practice and not recommended.^7^

Treatment options for the situation described would include bypass of the thrombosed cephalic vein from the brachial artery to most proximal site of the thrombus; but in this case the thrombus extended as far as the left subclavian vein.^8^ In cases of central occlusion, endovascular thrombolysis techniques can be used in acute cases with low doses of urokinase, tissue plasminogen activator (tPA), combined with thromboaspiration or stent angioplasty, when there is central stenosis or cephalic arch stenosis causing hypertension.^9^ These techniques offer good results when early signs of access failure are detected, which probably could have occurred with the case described before the outcome of complete and extended thrombosis of the cephalic vein.^5^^,^ 10 Another reason for using the contralateral limb was the fact that the patient was subject to limitations affecting exercises to stimulate maturation of a possible fistula, because movements of the affected hand were painful.^3^

The literature recommends that side-to-end anastomoses be used, to prevent development of venous hypertension after creation of arteriovenous access for hemodialysis; that large caliber collaterals, with diameters similar to the primary venous return vein, should be ligated along the first 10 cm of the AVF; and that limbs with a history of subclavian access be avoided.^4^^,^ 11 ^,^ 12

## CONCLUSIONS

Treatment for venous ulcers secondary to venous hypertension should be preceded by careful investigation with vascular ultrasound on a case-by-case basis. In the case described here, the decision was taken to create a new AVF in the contralateral limb and ligate the previous AVF to achieve improvement of venous hypertension, healing of the ulcers, and good maturity of the new AVF. Vascular echography proved to be of fundamental importance to treatment, given the rare finding of greater venous flow steal to the distal segment.
