# Supplementary material for: Ulcers of the fingers and dorsum of the left hand caused by venous hypertension after construction of a brachiocephalic arteriovenous fistula: case report
Source: J Vasc Bras. 2019 Oct 23;18:e20190008. doi: 10.1590/1677-5449.190008 (PMC6822961; doi:10.1590/1677-5449.190008)
Supplement: Translation

# Translation: Úlceras de quirodáctilos e dorso de mão esquerda ocasionadas por hipertensão venosa após confecção de fístula arteriovenosa braquiocefálica: relato de caso

**Authors:** Aline Ioshie Akamine Asari, Daiane Cristina Ferreira Damasceno, Nathália Araújo de Almeida, Hícaro do Carmo Moreira, Ricardo André Viana Barros, Francisco Alberto Bezerra Ximenes

**Affiliations:** 1 Hospital de Base do Distrito Federal – HBDF, Unidade de Cirurgia Vascular, Brasília, DF, Brasil.

**Keywords:** fístula arteriovenosa, diálise renal, insuficiência renal crônica, úlcera cutânea

## Abstract

Úlceras venosas decorrentes de hipertensão venosa por fístula arteriovenosa são raras. A investigação de sua etiologia é esclarecida pelo eco-Doppler vascular, que diferencia entre estenose de vasos centrais e sobrecarga hemodinâmica gerada pelo desenvolvimento de tributárias, a partir da veia responsável pela fístula arteriovenosa. Apresentamos um caso gerado por sobrecarga hemodinâmica em tributária, que desviou o fluxo principal da fístula distalmente ao membro. Optamos por realizar a ligação da fístula para tratamento das úlceras e confecção de nova fístula arteriovenosa em membro contralateral.

## INTRODUÇÃO

A hipertensão venosa em mão decorrente de fístula arteriovenosa (FAV) para hemodiálise em membro superior é considerada uma complicação rara. Séries de casos da literatura relatam uma taxa de incidência de 0,13 a 0,78%. Inserida nesse contexto, a ocorrência de úlcera por hipertensão venosa em membro superior é ainda mais rara, tendo sido encontrados por Debus et al. apenas 16 casos na literatura. As causas dessas úlceras podem ser FAV para hemodiálise, principalmente se realizada anastomose laterolateral, e má-formação arteriovenosa congênita ou lesão vascular traumática, as quais podem complicar com estenose de veia central ou incompetência valvular da veia de drenagem da FAV. A ecografia vascular auxilia no diagnóstico etiológico e guia o tratamento, que objetiva reduzir a pressão venosa, seja com confecção de desvio do deságue venoso, reconstrução completa da FAV ou ligadura da fístula^1^^,^ 2.

## DESCRIÇÃO DO CASO

Um paciente do sexo masculino, 45 anos, previamente hipertenso, vítima de acidente automobilístico havia 17 anos, foi identificado com hemotórax, sendo submetido a toracotomia e traqueostomia. Permaneceu internado em unidade de terapia intensiva por 105 dias, e nesse período fez uso de antimicrobianos nefrotóxicos, evoluindo com insuficiência renal crônica. Possuía FAV braquiocefálica em membro superior esquerdo que, após 10 anos de sua confecção, apresentou aumento de fluxo e úlceras no dorso da mão esquerda e no quarto e quinto quirodáctilos esquerdos (Figuras 1 e 2), que provocavam dor limitante para atividades manuais.

**Figura 1:** Membros superiores: face dorsal com edema e hipercromia de mão esquerda.

**Figura 2:** Mão esquerda: face palmar (A) com úlcera em face de quarto quirodáctilo esquerdo e face dorsal (B) com úlcera cicatrizada no dorso da mão e no quarto e quinto quirodáctilos esquerdos.

Foi realizado eco-Doppler vascular de FAV com identificação de anastomose pérvia. O fluxo arterial apresentava-se normal desde a artéria braquial até a radial e ulnar (Figura 3), além de direcionamento do fluxo, velocidades sistólicas e diastólicas, índices de resistência e demais aspectos ecográficos não compatíveis com roubo de fluxo.

**Figura 3:** Artéria radial esquerda pérvia e com fluxo trifásico. Velocidade de pico sistólico de 33,3 cm/s.

Portanto, pelos sinais clínicos e complementação ecográfica, o diagnóstico foi de hipertensão venosa por oclusão da veia de drenagem no braço (cefálica) e hiperdesenvolvimento de colateral descendente (Figura 4). O fluxo da FAV desviado para o ramo marginal era de 446 mL/min, chegando a 2.763 mL/min no nível do punho (Figura 5).

**Figura 4:** Desenho esquemático da fístula arteriovenosa braquiocefálica esquerda com trombo na veia cefálica e roubo de fluxo para o ramo marginal.

**Figura 5:** Volume de fluxo no ramo marginal: proximal (A) com 446 mL/min; distal e ao nível do punho (B) com 2.763 mL/min.

Detectada a complicação em curso, seguiu-se o exame com mapeamento para detecção do melhor ponto para realizar uma nova FAV, a qual foi braquiobasílica direita com superficialização. O paciente permaneceu dialisando pela FAV do membro superior esquerdo até a maturação da nova FAV, não sendo necessário submetê-lo a colocação de cateter.

Após a maturação da FAV, foi realizada a ligadura e secção da tributária do membro esquerdo no ponto mais proximal possível (no cotovelo). A melhora do edema e a cicatrização dos ferimentos nos dedos ocorreram em uma semana de pós-operatório. Na segunda semana, o paciente retornou ao ambulatório já com úlceras cicatrizadas. No seguimento de dois meses da alta hospitalar, já apresentava retorno da funcionalidade da mão esquerda (Figura 6). A fístula braquiobasílica direita tinha bom frêmito e fluxo à hemodiálise, e permaneceu apenas a coloração mais escura no membro direito.

**Figura 6:** Segunda semana de pós-operatório da ligadura da fístula arteriovenosa.

## DISCUSSÃO

A hipertensão venosa é um importante problema nos pacientes dialíticos, pois gera edema significativo do membro acometido e compromete a FAV. Consiste em uma das muitas possíveis complicações de longo prazo da confecção da FAV para hemodiálise. Desenvolve-se por um dos dois motivos a seguir: por consequência de estenose de vasos centrais ou por sobrecarga hemodinâmica gerada pelo desenvolvimento de tributárias oriundas da veia escolhida para a anastomose com a artéria^3^.

Similar à hipertensão venosa dos membros inferiores, a qual pode ser sequela de uma trombose venosa profunda ou de uma insuficiência valvar primária, carrega sintomas que, por vezes, causam limitações como edema e ulcerações na pele, assim como a repulsa quanto ao aspecto estético. Em situações extremas, tais ulcerações podem sofrer contaminação secundária e potencializar os danos. Com relação aos membros superiores, poderá haver limitações para atividades cotidianas manuais e até mesmo implicações para a utilização dos acessos vasculares ipsilaterais.

Tal sintomatologia pode ser confundida com a síndrome do roubo de fluxo, mas a ecografia vascular está indicada para realizar a diferenciação e fornecer detalhes para a estratégia cirúrgica de correção^3^. Conforme descrito, o suprimento arterial distal do membro relatado encontrava-se normal e, à ecografia, verificou-se que o paciente não possuía veias perfurantes para o sistema venoso profundo partindo da tributária principal e descendente, o que pode potencializar os sinais de hipertensão venosa.

Um estudo realizado por Nascimento e Riella a respeito das complicações relacionadas às fístulas evidenciou que a mais prevalente era trombose (80% dos casos), a qual ocorre devido à estenose frequentemente da anastomose venosa, por hiperplasia intimal^4^^,^ 5. No caso aqui apresentado, após oclusão da veia cefálica em braço, houve o desenvolvimento de fluxo numa colateral, a partir do cotovelo em direção à mão, que por muito tempo foi a única via de punção, mostrando-se útil. No entanto, a utilização rotineira dessa colateral como via de punção acabou por desenvolver hipertensão venosa com sintomas limitantes ao paciente. Tal caso levanta a polêmica de quando e por quanto tempo se pode usar colaterais como via de acesso para hemodiálise, uma vez que são mais tendenciosas a complicações por apresentarem calibre menor e se dirigirem para tecidos distais.

Currier et al.^6^, em 1986, sistematizaram a nomenclatura dos procedimentos relacionados às FAVs e desenvolveram uma classificação do grau de severidade da hipertensão venosa, conforme a seguir: 0, nenhuma severidade; 1, leve, caracterizada por sintomas mínimos, descoloração e discreto edema da extremidade; 2, moderada, caracterizada por desconforto intermitente e edema severo, sendo a intervenção usualmente necessária; e 3, grave, caracterizada por desconforto persistente com hiperpigmentação da pele, edema persistente e severo e úlcera venosa, sendo a intervenção mandatória.

O paciente do caso relatado enquadrava-se no grau 3 de severidade, com necessidade inquestionável de intervenção. Foi realizada confecção de nova FAV, seguida de ligadura da antiga FAV. No entanto, tal sequência pode ser inviável na presença de infecções nas úlceras resultantes de hipertensão venosa, bem como pode promover infecção, por via hematogênica, de possíveis cateteres. As bacteremias são problemas comuns em pacientes em hemodiálise, chegando a ser 26 vezes mais frequente do que na população geral, e a exposição cirúrgica durante as infecções é uma prática perigosa e não recomendada^7^.

As opções de tratamento para a situação descrita incluem o desvio da veia cefálica trombosada entre a artéria braquial e o local mais proximal ao trombo; porém, o trombo se estendia até a veia subclávia esquerda^8^. Em casos de oclusão central, pode-se utilizar técnicas endovasculares de trombólise em casos agudos com baixas doses de uroquinase, ativador do plasminogênio tecidual (tPA), combinada com tromboaspiração ou angioplastia com stents, quando existem estenoses centrais ou do arco cefálico causando a hipertensão^9^. Tais técnicas apresentam bons resultados quando são detectados sinais precoces de falha do acesso, o que provavelmente poderia ter acontecido com o caso descrito anteriormente ao desfecho da trombose completa e longa da veia cefálica^5^^,^ 10. Outro motivo para utilizar o membro contralateral foi o fato de o paciente apresentar limitações para exercícios que estimulam a maturação de uma possível fístula, pois os movimentos com a mão afetada eram dolorosos^3^.

A literatura recomenda que, para prevenção do desenvolvimento de hipertensão venosa após a confecção do acesso arteriovenoso para hemodiálise, sejam realizadas anastomoses terminolaterais; que ramos colaterais calibrosos, com diâmetro semelhante ao da veia de drenagem principal, sejam ligados nos primeiros 10 cm da FAV; e que se evite utilizar membros com histórico de acesso em subclávia^4^^,^ 11 ^,^ 12.

## CONCLUSÃO

O tratamento das úlceras venosas decorrentes de hipertensão venosa deve ser precedido de minuciosa investigação ecográfica vascular e individualizada. No caso apresentado, optou-se pela confecção no membro contralateral e ligadura da FAV prévia visando melhora da hipertensão venosa, cicatrização das úlceras e melhor maturação da nova FAV. A ecografia vascular realizada teve papel fundamental no tratamento dado o achado raro de desvio do maior fluxo venoso para o segmento distal.
